# Competing risk model to determine the prognostic factors and treatment strategies for elderly patients with glioblastoma

**DOI:** 10.1038/s41598-021-88820-5

**Published:** 2021-04-29

**Authors:** Zhuo-yi Liu, Song-shan Feng, Yi-hao Zhang, Li-yang Zhang, Sheng-chao Xu, Jing Li, Hui Cao, Jun Huang, Fan Fan, Li Cheng, Jun-yi Jiang, Quan Cheng, Zhi-xiong Liu

**Affiliations:** 1 Department of Anesthesiology, Xiangya Hospital, Center South University, Changsha, Hunan People’s Republic of China; 2 Department of Neurosurgery, Xiangya Hospital, Center South University, Changsha, Hunan People’s Republic of China; 3 National Clinical Research Center for Geriatric Disorders, Xiangya Hospital, Central South University, Changsha, Hunan People’s Republic of China; 4 Xiangya Cancer Center, Xiangya Hospital, Central South University, Changsha, People’s Republic of China; 5 Key Laboratory of Molecular Radiation Oncology of Hunan Province, Changsha, China; 6 Department of Rehabilitation, Second Xiangya Hospital, Central South University, Changsha, Hunan People’s Republic of China; 7 Department of Psychiatry, The Second People’s Hospital of Hunan Province, The Hospital of Hunan University of Chinese Medicine, Changsha, Hunan People’s Republic of China; 8 Center for Medical Genetics and Hunan Provincial Key Laboratory of Medical Genetics, School of Life Sciences, Central South University, Changsha, People’s Republic of China; 9 Department of Emergency, Fengyang County Hospital of Traditional Chinese Medicine, Anhui, People’s Republic of China; 10 Aier School of Ophthalmology, Central South University, Changsha, People’s Republic of China

**Keywords:** Cancer epidemiology, Cancer therapy, CNS cancer

## Abstract

The prognostic factors and optimal treatment for the elderly patient with glioblastoma (GBM) were poorly understood. This study extracted 4975 elderly patients (≥ 65 years old) with histologically confirmed GBM from Surveillance, Epidemiology and End Results (SEER) database. Firstly, Cumulative incidence function and cox proportional model were utilized to illustrate the interference of non-GBM related mortality in our cohort. Then, the Fine-Gray competing risk model was applied to determine the prognostic factors for GBM related mortality. Age ≥ 75 years old, white race, size > 5.4 cm, frontal lobe tumor, and overlapping lesion were independently associated with more GBM related death, while Gross total resection (GTR) (HR 0.87, 95%CI 0.80–0.94, *P* = 0.010), radiotherapy (HR 0.64, 95%CI 0.55–0.74, *P* < 0.001), chemotherapy (HR 0.72, 95%CI 0.59–0.90, *P* = 0.003), and chemoRT (HR 0.43, 95%CI 0.38–0.48, *P* < 0.001) were identified as independently protective factors of GBM related death. Based on this, a corresponding nomogram was conducted to predict 3-, 6- and 12-month GBM related mortality, the C-index of which were 0.763, 0.718, and 0.694 respectively. The calibration curve showed that there was a good consistency between the predicted and the actual mortality probability. Concerning treatment options, GTR followed by chemoRT is suggested as optimal treatment. Radiotherapy and chemotherapy alone also provide moderate clinical benefits.

## Introduction

Glioblastoma (GBM) are the most common malignant tumor in central nerves system (CNS) with dismal prognosis. The incidence of GBM is increasing with advancing age and it is reported that more than half of the patients with GBM were older than 65 years old^1^. Also, older age is a validated predictor for poor clinical outcome for patients with GBM^2,3^. However, the prognostic factors and optimal therapeutic strategy of elderly (age ≥ 65) with GBM are still controversial. Several meta-analysis studies of the elderly with GBM suggested that maximal surgical resection and postoperative ChemoRT provided promising clinical benefits for the elderly with GBM^4–6^. But there is still no large-scale retrospective study to validate the conclusions extracted from small-scale single-center clinical studies. Also elderly patients who died from non-GBM related causes such as cardiovascular disease and stroke will result in non-negligible competing risk bias^7,8^. But as far as I am concerned, all existed studies focused on elderly patients with GBM didn’t take this into consideration. Therefore, the current comprehension of the elderly with GBM is still insufficient and imprecise.

Cox proportional hazard model and Kaplan–Meier survival curve are the conventional analytic methods to determine the prognostic factors, while cumulative incidence function (CIF) curve and Fine-Gray competing risk model are more suitable for elderly patients, which can eliminate the interference of competing risk bias^9^. We took advantage of Surveillance, Epidemiology and End Results (SEER) database to include 4975 elderly patients with histologically confirmed GBM, which provided a detailed record of causes of death. This is the first large-scale population-based study based on competing risk model to determine prognostic factors and optimal therapeutic options for this frail patient group.

## Method

### Study population

**Figure 1:**
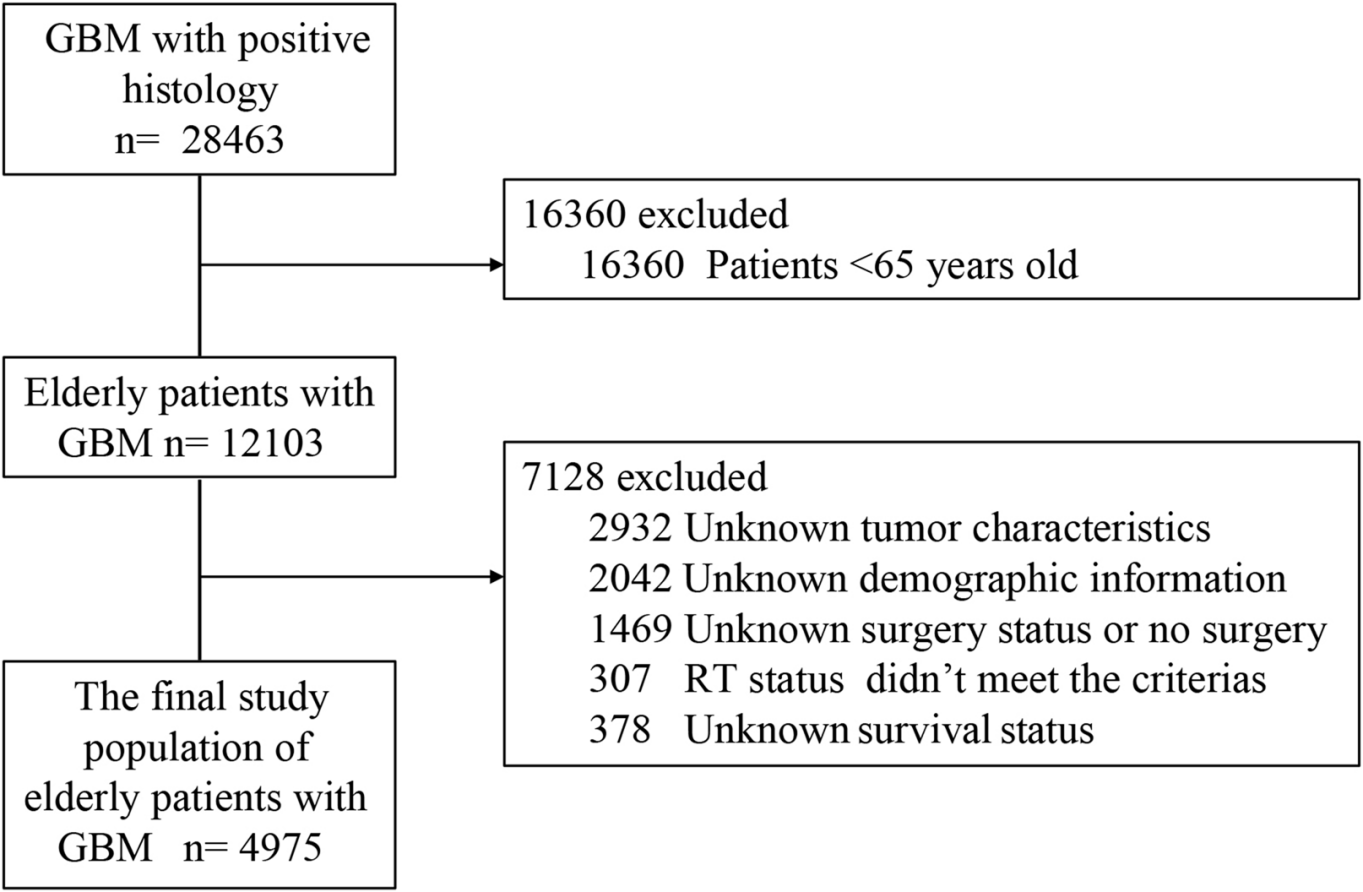
Flowchart of elderly with glioblastoma selection.

The National Cancer Institute sponsored SEER database was searched to identify the elderly with histologically confirmed GBM between 2007 and 2016. The SEER database provides de-identified information including cancer incidence, patients’ demographic, tumor characteristics, treatment options, and survival outcomes. All patients with histologically confirmed GBM were firstly included. Then, patients with age < 65 years old were excluded. Patients with unknown tumor characteristics, demographic information, and survival status were excluded. Concerning radiotherapy, only the patients treated with beam radiation after surgery or no/unknown were included. Regarding surgical resection, the patients treated with no surgery (code 00) were excluded. The final study population strictly included patients treated with biopsy (code 20), subtotal resection (STR, code 21), or gross total resection (GTR, codes 30, 40, and 55) (Fig. 1).

### Covariates included

The following patient data was obtained for the analysis: age group (< 75 years old, ≥ 75 years old ), sex (female, male,) race (other, white, and black), marital status (single, married/partner, unmarried/divorced, and widowed), insurance status (privately insured, Medicaid, and uninsured), size (size ≤ 5.4 cm, size > 5.4 cm, the best cut-off value was defined according to X-tile software), metastasis (yes, no), primary site (temporal, cerebrum, frontal, parietal, occipital, ventricle, cerebellum, brainstem, and overlapping lesion). Concerning treatment options, extent of surgery (biopsy, STR GTR) and adjuvant therapy (radiotherapy, chemotherapy, ChemoRT, no/unknown) were analyzed (Table 1).

**Table 1:** Patient demographics, tumor characteristics and treatment options of 4975 elderly with glioblastoma.

Characteristics	All	Biopsy (%)	GTR (%)	STR (%)	*P* value
**Population size**	4975	1191 (23.9)	2558 (51.4)	1226 (24.5)	
**Age group**					< 0.001^†^
< 75	3285	741 (22.6)	1753 (53.4)	791 (24.0)	
≥ 75	1690	450 (26.6)	805 (47.6)	435 (25.8)	
**Sex**					0.143
Female	2202	542 (24.2)	1171 (52.5)	523 (23.3)	
Male	2733	649 (23.8)	1381 (50.5)	703 (25.7)	
**Race**					0.003^†^
Black	207	65 (31.4)	103 (49.8)	39 (18.8)	
Other	231	53 (22.9)	103 (44.6)	75 (32.5)	
White	4537	1073 (23.7)	2352 (51.8)	1112 (24.5)	
**Marital status**					0.007^†^
Divorced/separated	410	108 (26.3)	204 (49.8)	98 (23.9)	
Married/partner	3343	745 (22.3)	1754 (52.5)	844 (25.2)	
Single	414	117 (28.3)	192 (46.4)	105 (25.4)	
Widowed	808	221 (27.4)	408 (50.5)	179 (22.1)	
**Insurance**					0.047^†^
Privately insured	4600	1096 (23.8)	2370 (51.5)	1134 (24.7)	
Medicaid	348	85 (24.4)	182 (52.3)	81 (23.3)	
Uninsured	27	10 (37.0)	6 (22.2)	11 (40.7)	
**Primary site**					< 0.001^†^
Temporal	1595	321 (20.1)	883 (55.4)	391 (24.5)	
Cerebrum	70	47 (67.1)	8 (11.4)	15 (21.5)	
Frontal	1406	329 (23.4)	728 (51.8)	349 (24.8)	
Parietal	899	232 (25.8)	457 (50.8)	210 (23.4)	
Occipital	273	57 (20.9)	156 (57.1)	60 (22.0)	
Ventricle	10	4 (40.0)	4 (40.0)	2 (20.0)	
Cerebellum	29	8 (27.6)	16 (55.2)	5 (17.2)	
Brainstem	1	1 (100)	0 (0.0)	0 (0.0)	
Overlapping	692	192 (27.8)	306 (44.2)	194 (28.0)	
**Size**					< 0.001^†^
> 5.4 cm	1337	300 (21.8)	6661 (48.4)	411 (29.8)	
≤ 5.4 cm	3598	891 (24.8)	1892 (52.6)	815 (22.7)	
**Metastasis**					0.014^†^
No	4928	1178 (23.9)	2543 (51.6)	1207 (24.5)	
Yes	47	13 (27.7)	15 (31.9)	19 (40.3)	
**Adjuvant therapy**					< 0.001^†^
No/unknown	861	241 (28.0)	430 (49.9)	190 (22.1)	
Radiotherapy	530	153 (28.9)	252 (47.5)	125 (23.6)	
Chemotherapy	142	36 (25.4)	79 (55.6)	27 (19.0)	
ChemoRT	3442	761 (22.1)	1797 (52.1)	884 (25.7)	

*GTR* gross total resection, *STR* subtotal resection.

^†^*P* < 0.05, statistically significant.

### Statistical analyses

The baseline patient characteristics were compared among patients treated with different extent of surgery by Chi-square test. GBM related death and non-GBM related death were defined as two competing events. Firstly, Cumulative incidence function (CIF) was plotted to show the probability of GBM related mortality (curve1) and non-GBM related mortality (curve2), Gray’s test was applied to analyze the differences between groups. Secondly, the univariate and multivariable Cox proportional regression were used to reveal the relationship between the covariates and non-GBM related mortality. Thirdly, univariate and multivariable competing risk analyses were utilized to identify prognostic factors for GBM related mortality to eliminate the interference of competing events^8,10^. Finally, a corresponding nomogram based on the identified prognostic factors were then conducted to predict 3-month, 6-month, and 12-month GBM related mortality using the R packages rms and mstage. The discrimination performance of the nomogram was evaluated by concordance index (C-index) and a calibration curve was plotted via a bootstrap method with 1000 resamples to estimate the consistency between the predicted and the actual survival probability^11^. All statistical analyses were performed in R version 3.5.1 (http://www.r-project.org/). R packages cmprsk were used for the competing risk analysis/, rms and mstage were utilized to build the model and nomogram. pec was applied to evaluate the performance of the nomogram. *P* < 0.05 was considered statistically significant.

## Results

### Characteristics of the study population

4975 patients were included. The median survival month of the cohort was 8 months. The 3-month, 6-month, and 12-month overall survival rates were 75.6%, 56.9%, and 34.2% respectively. 2558 patients (51.4%) received GTR, 1226 (24.5%) patients got STR, and 1191patients (23.9%) treated with biopsy only. The OS rates of biopsy, STR, and GTR group were 7.9%, 16.6%, and 14.1% respectively. Most preoperative covariates, except sex (*P* = 0.143) were statistically different among groups of biopsy, STR, and GTR. More patients in < 75 years old group received GTR than ≥ 75 years old group. (53.4% vs. 47.6%, *P* < 0.001). Patients with white race and patients grouped as married/partner had relatively higher GTR rate (51.8% and 52.5%, respectively). Patients with tumor size ≤ 5.4 cm had a significantly higher GTR rate (52.6% vs. 48.4%, *P* < 0.001). Concerning tumor site, more than 50% of tumors located in occipital lobe, temporal lobe, cerebellum, frontal lobe, and parietal lobe received GTR. (57.1%, 55.4%, 55.2%, 51.8%, and 50.8% respectively), while cerebrum and overlapping tumor had relatively lower GTR rates. (44.2% and 11.4% respectively). For brainstem and ventricular tumor, the population size is too small to get accurate data. (n = 1 and n = 10, respectively) (Table1).

### Interferences of non-GBM related death

**Figure 2:**
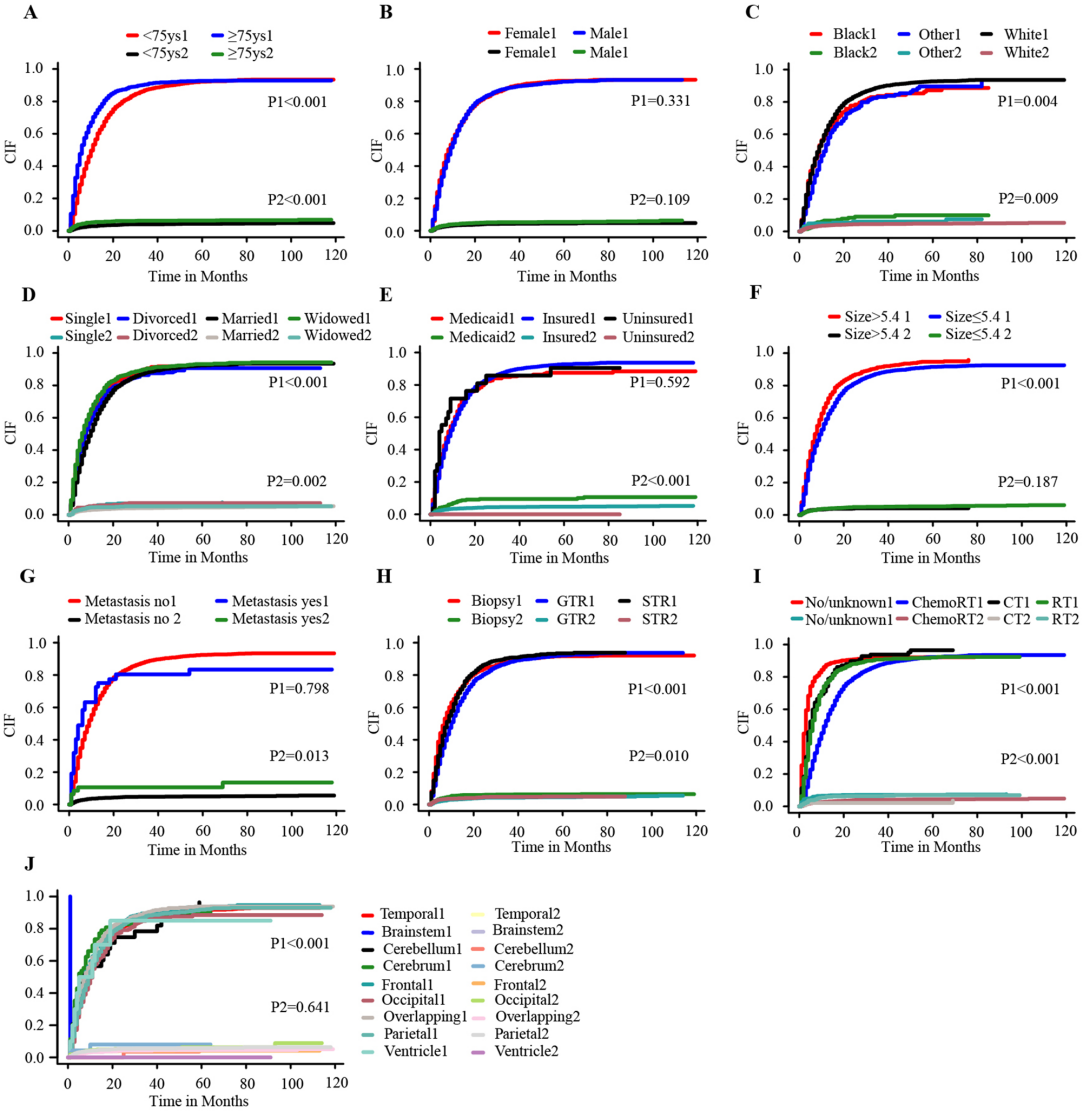
CIF curves for elderly with GBM by different variates. **(A),** Age group; **(B),** Sex; **(C),** Race; **(D)**, Marital status; **(E)**,Insurance status; **(F)**, Tumor size; (**G**), Metastasis; (**H**),Surgery (**I**), Adjuvant therapy; (**J**), Primary site. *Note*: P1 represents the difference of GBM related death. P2 represents the difference of non-GBM related death.

At the time of data collected, 13.2% (n = 659) of patients were alive, and 82.1% (n = 4082) of patients died from GBM, and 4.7% (n = 234) of patients died from competing causes. The result of CIF showed that patients in ≥ 75 years old, black race, divorced, Medicaid, with metastasis, biopsy, and no/unknown adjuvant therapy groups had significantly higher risk of death from non-GBM related death (Fig. 2). The results of univariate and multivariable cox analysis showed that < 75 years old, female, married, medicaid, GTR (HR 0.90, 95%CI 0.84–0.95, *P* < 0.001), Radiotherapy (HR 0.65, 95%CI 0.58–0.72, *P* < 0.001), chemotherapy (HR 0.61, 95%CI 0.53–0.70, *P* < 0.001), and chemoRT (HR 0.46, 95%CI 0.43–0.50, *P* < 0.001) had an independently significant association with less non-GBM related death (Table 2).

**Table 2:** Univariate and multivariable cox proportional analyses for non-GBM related death in elderly patients with glioblastoma.

	Univariate analysis		Multivariable analysis	
	HR (95%CI)	*P* value	HR (95%CI)	*P* value
**Age**				
< 75	1 [Reference]		1 [Reference]	
≥ 75	2.17 (1.67–2.81)	< 0.001^†^	1.95 (1.48–2.60)	< 0.001^†^
**Sex**				
Female	1 [Reference]		1 [Reference]	
Male	1.24 (0.95–1.61)	0.105	1.45 (1.09–1.93)	0.009^†^
**Race**				
Other	1 [Reference]		1 [Reference]	
Black	1.59 (0.79–3.19)	0.194	1.45 (0.71–2.98)	0.306
White	0.81 (0.47–1.39)	0.446	0.85 (0.49–1.49)	0.586
**Marital status**				
Single	1 [Reference]		1 [Reference]	
Divorced/separated	0.90 (0.53–1.54)	0.706	1.14 (0.66–1.95)	0.634
Married/partner	0.51 (0.34–0.77)	0.002	0.61 (0.40–0.94)	0.023^†^
Widowed	0.74 (0.45–1.21)	0.244	0.74 (0.45–1.21)	0.244
**Insurance status**				
Insured	1 [Reference]		1 [Reference]	
Medicaid	0.44 (0.30–0.64)	< 0.001^†^	0.53 (0.36–0.79)	< 0.002^†^
Uninsured	/	/	/	/
**Primary site**				
Temporal	1 [Reference]		1 [Reference]	
Brainstem	/	/	/	/
Cerebellum	0.62 (0.09–4.42)	0.630^†^	0.43 (0.06–3.15)	0.409
Cerebrum	1.97 (0.79–4.85)	0.143	1.49 (0.59–3.73)	0.400
Frontal	0.87 (0.62–1.23)	0.445	0.85 (0.60–1.19)	0.341
Occipital	1.09 (0.64–1.87)	0.755	1.11 (0.65–1.90)	0.710
Overlapping	0.93 (0.60–1.42)	0.722	0.90 (0.59–1.39)	0.641
Parietal	1.14 (0.80–1.63)	0.477	1.05 (0.73–1.51)	0.792
Ventricle	/	/	/	/
**Size**				
≤ 5.4	1 [Reference]		1 [Reference]	
> 5.4	1.07 (0.79–1.46)	0.652	1.18 (0.86–1.61)	0.300
**Metastasis**				
No	1 [Reference]		1 [Reference]	
Yes	3.01 (1.32–6.87)	0.009	2.33 (0.99–5.47)	0.052
**Surgery**				
Biopsy	1 [Reference]		1 [Reference]	
GTR	0.60 (0.45–0.81)	0.001^†^	0.67 (0.49–0.90)	0.010^†^
STR	0.75 (0.53–1.07)	0.116	0.87 (0.61–1.25)	0.461
**Adjuvant therapy**				
No/unknown	1 [Reference]		1 [Reference]	
Both	0.23 (0.16–0.31)	< 0.001^†^	0.27 (0.20–0.37)	< 0.001^†^
Chemotherapy	0.24 (0.09–0.65)	0.005^†^	0.24 (0.09–0.68)	0.007^†^
Radiotherapy	0.56 (0.37–0.85)	0.007^†^	0.55 (0.36–0.84)	0.006^†^

*GTR* gross total resection, *STR* subtotal resection.

^†^*P* < 0.05, statistically significant;

### Prognostic factors of GBM related death

CIF revealed that patients in ≥ 75 years old, white race, size > 5.4 cm, and overlapping lesion groups had significantly higher probability of GBM related death, while married/partner, GTR, and chemoRT were significantly associated with less GBM related death (Fig. 2). Further univariate and multivariable competing risk analyses were conducted to identify independent prognostic factors for GBM related death. The result showed that ≥ 75 years old, white race, frontal lobe tumor, overlapping tumor, brainstem tumor, size > 5.4 cm were identified as independently risk factors of GBM related death. Regarding treatment options, GTR (HR 0.87, 95%CI 0.80–0.94, *P* = 0.010), radiotherapy (HR 0.64, 95%CI 0.55–0.74, *P* < 0.001), chemotherapy (HR 0.72, 95%CI 0.59–0.90, *P* = 0.003), and chemoRT (HR 0.43, 95%CI 0.38–0.48, *P* < 0.001) were identified as independently protective factors for GBM related death. STR showed no significant relation with GBM related death in both univariate (HR 0.96, 95%CI 0.88–1.05, *P* = 0.35) and multivariable (HR 1.03, 95%CI 0.93–1.13, *P* = 0.60) competing risk analysis (Table 3).

**Table 3:** Univariate and multivariable competing risk analyses for GBM related death in elderly patients with glioblastoma.

	Univariate analysis		Multivariable analysis	
	HR (95%CI)	*P* value	HR (95%CI)	*P* value
**Age**				
< 75	1 [Reference]		1 [Reference]	
≥ 75	1.41 (1.32–1.51)	< 0.001^†^	1.22 (1.14–1.32)	< 0.001^†^
**Sex**				
Female	1 [Reference]		1 [Reference]	
Male	0.97 (0.92–1.03)	0.37	1.01 (0.94–1.08)	0.87
**Race**				
Other	1 [Reference]		1 [Reference]	
Black	1.13 (0.92–1.39)	0.260	1.13 (0.89–1.42)	0.33
White	1.27 (1.11–1.47)	< 0.001^†^	1.33 (1.14–1.54)	< 0.001^†^
**Marital status**				
Single	1 [Reference]		1 [Reference]	
Divorced/separated	0.91 (0.79–1.07)	0.280	0.95 (0.80–1.12)	0.51
Married/partner	0.86 (0.78–1.07)	0.016	0.93 (0.82–1.06)	0.26
Widowed	1.17 (0.98–1.28)	0.110	1.07 (0.92–1.24)	0.41
**Insurance status**				
Insured	1 [Reference]		1 [Reference]	
Medicaid	1.04 (0.91–1.18)	0.59	1.11 (0.96–1.29)	0.15
Uninsured	1.18 (0.71–2.00)	0.52	1.27 (0.82–1.98)	0.27
**Primary site**				
Temporal	1 [Reference]		1 [Reference]	
Brainstem	16.5 (14.8–18.5)	< 0.001^†^	10.1 (8.54–11.9)	< 0.001^†^
Cerebellum	1.01 (0.69–1.47)	0.98	0.95 (0.56–1.61)	0.85
Cerebrum	1.28 (0.95–1.74)	0.11	1.14 (0.79–1.64)	0.48
Frontal	1.17 (1.08–1.26)	< 0.001^†^	1.17 (1.08–1.27)	< 0.001^†^
Occipital	0.92 (0.80–1.05)	0.21	0.93 (0.81–1.08)	0.37
Overlapping	1.18 (1.17–1.30)	0.007^†^	1.18 (1.07–1.31)	0.002^†^
Parietal	1.05 (0.47–2.37)	0.90	1.05 (0.95–1.15)	0.34
Ventricle	/	/	0.93 (0.45–1.93)	0.85
**Size**				
≤ 5.4	1 [Reference]		1 [Reference]	
> 5.4	1.23 (1.15–1.31)	< 0.001^†^	1.19 (1.10–1.28)	< 0.001^†^
**Metastasis**				
No	1 [Reference]		1 [Reference]	
Yes	1.07 (0.70–1.63)	0.75	0.91 (0.58–1.42)	0.68
**Surgery**				
Biopsy	1 [Reference]		1 [Reference]	
GTR	0.82 (0.76–0.89)	< 0.001^†^	0.87 (0.80–0.94)	0.010^†^
STR	0.96 (0.88–1.05)	0.35	1.03 (0.93–1.13)	0.60
**Adjuvant therapy**				
No/unknown	1 [Reference]		1 [Reference]	
Both	0.41 (0.36–0.46)	< 0.001^†^	0.43 (0.38–0.48)	< 0.001^†^
Chemotherapy	0.70 (0.56–0.86)	< 0.001^†^	0.72 (0.59–0.90)	0.003^†^
Radiotherapy	0.63 (0.54–0.72)	< 0.001^†^	0.64 (0.55–0.74)	< 0.001^††^

*GTR* gross total resection, *STR* subtotal resection.

^†^*P* < 0.05, statistically significant.

### Competing risk nomogram

**Figure 3:**
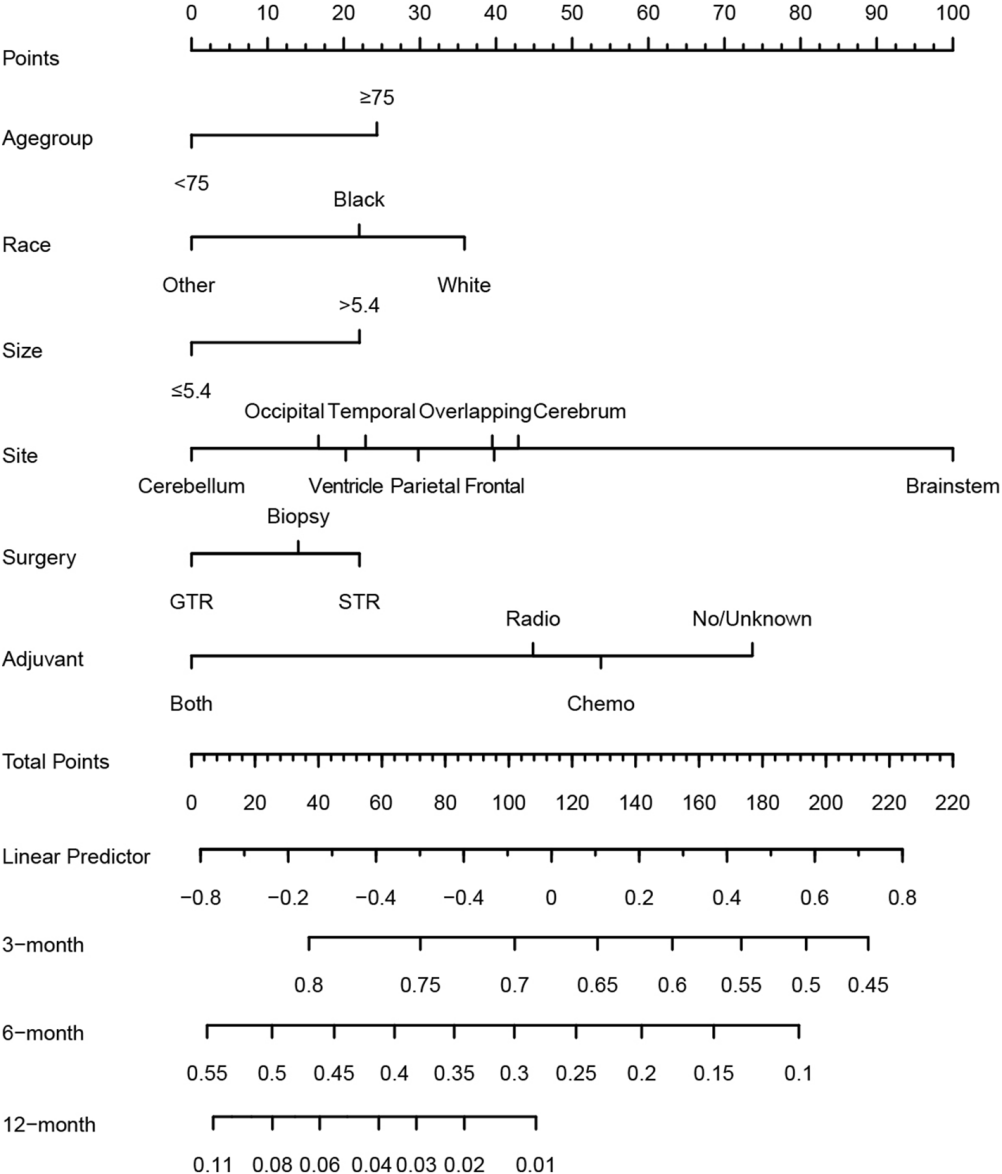
Competing risk nomogram to predict 3-, 6-, and 12-month GBM related mortality of the elderly patients with GBM.

**Figure 4:**
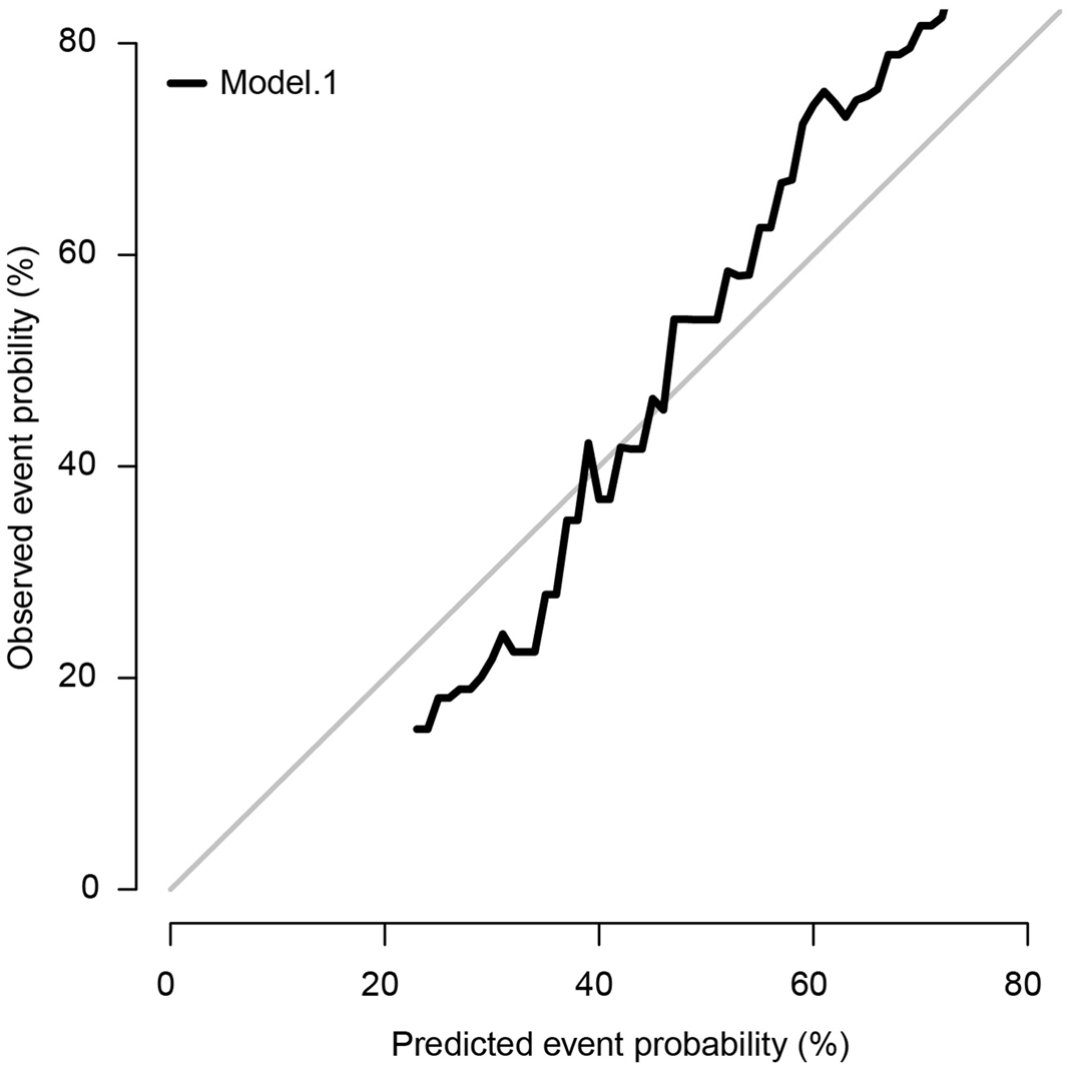
The calibration curve to show the consistency between the nomogram-predicted probabilities and actual observations.

Identified predictors including age group, sex, surgery, adjuvant, size, and site were integrated to develop the prognostic competing risk nomogram to predict the 3-month, 6-month, and 12-month GBM related mortality with C-index of 0.763, 0.718, and 0.694 respectively, which showed relative good discriminative. Site and adjuvant therapy were the top two strong predictors (Fig. 3). Calibration plots showed good agreement between the nomogram-predicted probabilities and actual observations (Fig. 4).

## Discussion

When patients with GBMs died from causes e.g. infections, injury, accidents, cardiovascular or cerebrovascular events, these deaths are competing risks. This phenomenon was much more frequently observed in the elderly population. Analysis based on OS results in an upward bias in the estimate of incidence, while the cause-specific cox model will underestimate the incidence as it treats non-GBM related death as censored observations. In our study cohort, the median survival month is 8 months, and 4.7% (n = 234) of patients died from competing causes. Although the percentage of non-GBM related death is not high, it caused non-negligible interference. The result of CIF and cox proportional analysis for non-GBM death showed that several covariates including extent of surgery and adjuvant therapy had a significant association with non-GBM related death. These results indicated that the Fine-Gray competing risk model was of great necessity to eliminate bias, especially for determining the prognostic value of GTR and adjuvant therapy in this study.

Regarding patient demographics, older age and white race were identified as independent risk factors of GBM related death. Consistently, a study of 273 elderly patients with GBM reported that the OS month was significantly higher in the 65–74 years group compared with the ≥ 75 years group (9.8 ± 10.8 vs. 5.2 ± 5.2 months, *P* = 0.0004)^12^. Another large-scale study including 150,631 patients with GBM reported that Asian and Pacific Islanders (API), which were defined as other races in this study, had the best prognosis^13^. Notably, there were studies reported that female and married were independently protective factors for CSS and OS^14,15^. However, our results revealed that female and married significantly related to lower risks non-GBM related death but not GBM related death. A possible explanation was that married patients might have better economic status, social support, and psycho-oncology services. And cardiovascular and cerebrovascular events were more commonly observed in male patients^16,17^. These disparities also indicated the value of the competing risk model in this study. Concerning tumor characteristics, frontal lobe tumor, overlapping tumor, brainstem tumor, and size > 5.4 cm were identified as independent risk factors of GBM related death smaller tumor size usually represented lower invasiveness and were more accessible to GTR^18^. Patients with tumor size ≤ 5.4 cm had a significantly higher GTR rate (52.6% vs. 48.4%, *P* < 0.001) in our cohort. Frontal lobe tumor, brainstem tumor, and overlapping lesion tumor reported having worse prognosis due to its infiltrative nature or critical anatomical position^19,20^.

GTR was identified independently related to decreased risks of non-GBM related death, which indicated that patients who received GTR may have a relatively better general condition. At the same time, GTR was proved to provide significantly protective effects from GBM related death after eliminating the competing risk bias. Consistently, most studies reported that GTR was superior to biopsy only with regards to OS^4,12,21,22^. However, there is still no consensus about the effects of STR. Our analyses revealed STR compared with biopsy had no significant association with decreased risk of GBM related death in univariate and multivariate competing analyses. A meta-analysis searched studies before 2014 concluded that the STR compared with biopsy experienced a significantly better OS of 2.55 months (95% CI 0.91–4.19, *P* = 0.002)^23^. However, this study didn’t adjust the confound effects of other covariates by multivariate regression. Another retrospective study including 124 patients (≥ 65 years) with GBM also reported that STR significantly improved OS compared with biopsy (Median 11.0 and 4.0 months, *P* < 0.02). However, the association between STR and improved OS was significant only in univariate analysis. Besides, a higher rate of complication was observed in STR compared with biopsy. (42.9% vs. 7.4%)^21^. In a recent retrospective study including 273 patients (65–84 years) with GBM, multivariate analysis revealed the benefit of STR over biopsy was significant only in the 65–75 years group (*P* = 0.01) but not the 75–84 years group (*P* = 0.081)^12^. According to our analysis and existed studies, GTR can provide valid clinical benefits for the elderly with GBM, which should be offered whenever safely possible. For patients couldn’t achieve GTR, biopsy rather than STR was generally preferred. More subgroup analyses were needed to determine which part of patients would benefit from STR.

ChemoRT showed the greatest protective effect for GBM related death (HR 0.46, 95%CI 0.43–0.50, *P* < 0.001) in our analyses. Consistent with our results, a population-based study including 5252 elderly with GBM (> 70 years, RT: n = 1389; chemoRT: n = 3863) reported that chemoRT was significantly associated with better OS than RT (HR 0.79, 95% CI 0.70–0.89, *P* < 0.001) on multivariate analysis^24^. Another retrospective study of 117 elderly with GBM reported the median OS months of patients who accessed chemoRT (n = 84) and those who didn’t (n = 33) were 11 and 5 months respectively. Another study including 616 patients with GBM from CGGA and TCGA databases also concluded that chemoRT could benefit both old and young patients, but old patients are currently less likely to choose it^2^. In our cohort, the percentage of the patient received chemoRT was decreasing with advancing age (76.3% in < 75 years old group and 59.5% in ≥ 75 years old group), meaning older patients had significantly less tolerance to adjuvant therapy. Recently, several studies have suggested that hypofractionated RT combined with CT provide similar or even better survival benefit compared with standard RT combined with CT, which is more tolerable for older patients^5,6,25^. Our results also revealed that either RT (HR 0.64, 95%CI 0.55–0.74, *P* < 0.001) or CT (HR 0.72, 95%CI 0.59–0.90, *P* = 0.003) also provide modest protective effect in GBM related death compared with no/unknown. Consistently, a clinical trial including 85 patients (≥ 70 years) with GBM reported that RT had a modest improvement in OS (HR 0.47, 95%CI 0.29–0.76, *P* = 0.002)^26^. Another clinical trial enrolling 373 patients (≥ 65 years) with GBM reported that CT was considered equally effective compared with RT (HR 1.09, 95%CI 0.84–1.42, *P* = 0.033)^27^. Both our analyses and existed studies suggested chemoRT was the optimal treatment for elderly patients with GBM. When it is intolerable, RT or CT alone were an alternative to provide moderate clinical benefits. Elderly GBM patients should receive customized adjuvant therapy whenever feasible.

This study has several limitations. Despite SEER database contains abundant medical records, it lacks some important information including neurological performance and function grading, tumor recurrence and quality of life, molecular signature and genetic alteration, detailed RT dosage and CT regimen, and type of treatment center. This study als0 has its strengths. This is the first large-scale retrospective study based on competing risk model to analyze elderly patients with GBM, which provides information with less bias and more strengths. And we highlighted the clinical benefits of GTR and adjuvant therapy for elderly patients with GBM.

## Conclusion

This study takes advantage of competing risk model to determine prognostic factors for the elderly patient with GBM accurately. Preoperative factors including ≥ 75 years old, white race, frontal lobe tumor, overlapping tumor, brainstem tumor, size > 5.4 cm were identified as risk factors. The competing risk nomogram was discriminative. GTR followed by chemoRT is suggested as optimal treatment. RT or CT alone also provides moderate clinical benefits. Elderly patients with GBM should receive customized adjuvant therapy whenever feasible.
